# Infrared–Depth Drogue Target Detection via Frequency-Domain Enhancement and Decoupled Gated Fusion

**DOI:** 10.3390/s26165247

**Published:** 2026-08-19

**Authors:** Baoshan Li, Haibo Wang, Dong Cao, Shilong Ji, Jinpei Xiao, Lanjin Lin

**Affiliations:** Computational Aerodynamics Institute, China Aerodynamics Research and Development Center, Mianyang 621000, China; wwwlibaoshan@163.com (B.L.); hbwang_cardc@163.com (H.W.); slji2020@alu.uestc.edu.cn (S.J.); xjp_1@mail.nwpu.edu.cn (J.X.); jenny_lin@alu.uestc.edu.cn (L.L.)

**Keywords:** multimodal detection, infrared–depth fusion, UAV drogue, wavelet enhancement, attention mechanism, edge computing

## Abstract

**Highlights:**

**What are the main findings?**

- Training-free AWIE enhances infrared images via frequency-domain adaptive modulation.
- Decoupled CGAF prevents feature confusion via independent cross-modal gating.

**What are the implications of the main findings?**

- AWIE-CGAF achieves 89.5% mAP@0.5 and 51.7 FPS on Jetson AGX Orin with only 13.5 M parameters.
- Results support real-time IR–D drogue detection on edge platforms.

**Abstract:**

High-precision drogue localization during terminal guidance is critical to close-range autonomous unmanned aerial vehicle (UAV) docking and hinges on infrared–depth (IR–D) multimodal detection. Yet, deploying such detection on airborne edge computing platforms faces severe challenges due to modal heterogeneity, feature redundancy, and real-time constraints. A lightweight IR–D fusion detection network, termed AWIE-CGAF, is proposed for airborne edge deployment, which integrates frequency-domain, physics-prior-driven input enhancement with decoupled gated attention-based adaptive feature fusion to achieve efficient multimodal detection. A training-free Adaptive Wavelet Image Enhancement (AWIE) module is designed to differentially modulate image structures and details in the frequency domain, improving the signal-to-noise ratio and feature discriminability. Concurrently, a Cross-Gated Attention Fusion (CGAF) module employs decoupled cross-modal attention with independent gating, preserving modality-specific features while dynamically selecting complementary information, mitigating redundancy and feature contamination. Experiments on the self-constructed Drogue Infrared–Depth (DIRD) dataset showed that AWIE-CGAF achieved 89.5% mAP@0.5 and 58.2% mAP@0.5:0.95 with 13.5 M parameters, while maintaining real-time inference at 51.7 FPS on a Jetson AGX Orin edge platform. Among the evaluated methods, the proposed framework achieved the highest detection accuracy while retaining real-time edge inference capability. These results support the feasibility of AWIE-CGAF for resource-constrained IR–D drogue perception.

## 1. Introduction

Autonomous UAV docking extends operational endurance and mission flexibility. In probe-and-drogue configurations, terminal engagement requires precise probe capture within narrow spatial and temporal margins. Flexible cable–drogue assemblies exhibit oscillations induced by carrier wake and atmospheric disturbances [1,2]. Reliable onboard perception of the drogue is therefore essential for relative navigation and closed-loop docking control. In practical environments, cloud clutter, strong glare, low target–background contrast, and partial occlusion can substantially degrade target observability. Meanwhile, airborne implementation imposes stringent constraints on inference latency, computational resources, and power consumption. Consequently, drogue perception requires a careful balance among detection accuracy, environmental robustness, and real-time deployment efficiency.

Vision-based drogue perception has progressively evolved from handcrafted geometric and appearance features toward learning-based and embedded architectures. Wang et al. [3] introduced convolutional neural networks (CNNs) for drogue detection using aerial refueling imagery. Ma et al. [4] incorporated structural constraints to support joint drogue detection and pose estimation, while Xu et al. [5] combined cascade AdaBoost with CNN classification to improve robustness in complex backgrounds. More recent studies have increasingly emphasized real-time onboard implementation. Bañuelos Garcia and Bani Younes [6] developed a deep-learning-based framework for real-time drogue navigation; Ma et al. [7] proposed a lightweight CNN architecture suitable for embedded deployment; and Gao et al. [8] integrated detection, tracking, prediction, and stereo measurement within an onboard perception system. Wu et al. [9], Zhai et al. [10], and Wen et al. [11] further improved drogue detection or pose estimation under scale variation, occlusion, and complex backgrounds. Despite these advances, most existing methods still rely predominantly on monocular appearance cues or predefined geometric structures and may therefore degrade when target texture, contrast, or visible structure becomes unreliable.

Introducing explicit depth information provides an effective means of reducing the geometric ambiguity inherent in appearance-only perception. Stereo vision has been employed to estimate drogue position and spatial orientation [12], while RGB–D frameworks have been investigated for target tracking and autonomous aerial docking [13]. Among active ranging technologies, Time-of-Flight (ToF) imaging is particularly attractive because it directly provides dense metric range measurements without relying on natural-texture correspondence. ToF sensors estimate depth from the propagation delay or phase relationship of actively emitted optical signals and generate regular two-dimensional depth maps that can be geometrically registered with image-based sensing modalities [14]. Such depth measurements explicitly encode range, contour, and scale information. Nevertheless, ToF sensing is also affected by multipath interference, weak signal returns, ambient illumination, surface reflectance, and local measurement corruption [14,15], demonstrating the potential of thermal-IR and ToF fusion for close-range perception with reduced dependence on external illumination and scene texture.

Infrared (IR) imaging provides a complementary sensing mechanism by capturing radiometric target characteristics with reduced dependence on visible light conditions. However, IR imagery often exhibits weak local texture and indistinct structural boundaries, particularly for small or low-contrast drogue targets. Conversely, ToF depth directly describes range and geometric structure but may become locally noisy or incomplete under unfavorable motion, reflectance, or ranging conditions. IR and ToF depth therefore exhibit complementary sensing characteristics and failure modes, motivating their joint exploitation in challenging docking environments. Rather than treating either modality as uniformly reliable, an effective IR–D perception system should selectively exploit their complementary radiometric and geometric cues. Previous studies have demonstrated the feasibility of registering thermal-IR and ToF measurements and jointly exploiting the two modalities for scene perception [16]. Recent ToF-based object-detection research has further shown that combining active IR and depth observations can outperform reliance on a single sensing channel [15]. These findings provide a physical basis for IR–D fusion in drogue detection.

While IR–D fusion has demonstrated efficacy in generic tasks such as pedestrian detection and semantic segmentation, targeted investigations for high-dynamic, mission-critical applications like cable-tethered drogue docking remain scarce. Vidas et al. [17] proposed a mask-based geometric calibration method for thermal-infrared cameras, laying the groundwork for cross-spectral alignment of ToF and thermal-infrared imaging systems. Hoegner et al. [16] fused ToF depth and thermal IR via fully automatic geometric calibration for scene segmentation and people detection, confirming illumination independence of the active-passive sensor pair in close-range applications. Yang et al. [15] fused active IR intensity and depth images from a single ToF camera for object detection, achieving 0.778 IoU in localization and 98.01% classification accuracy. Wang et al. [18] optimized small object detection using paired IR and depth images via a YOLOv5-based framework (Ultralytics YOLOv5, https://github.com/ultralytics/yolov5, accessed on 5 June 2026) enhanced with joint depth filtering and partial convolution. Complementary to these detection efforts, Achaibou et al. [19] proposed near-infrared-guided belief propagation to repair invalid ToF pixels, providing essential depth-quality enhancement for robust IR–D perception. Additionally, Sharma et al. [20] implemented depth-specific convolutions within a multi-task learning framework, demonstrating that IR–D from a single ToF camera achieves segmentation performance competitive with RGB-D baselines on semantic segmentation tasks, providing empirical support for the viability of IR–D as a standalone modality.

However, most of these approaches are developed for relatively generic recognition or scene-understanding tasks and do not directly address the operational characteristics of cable-tethered drogue perception. In this application, relative motion, scale variation, environmental interference, and modality-specific degradation may occur simultaneously. Moreover, fixed weighting, direct concatenation, or shallow fusion may propagate unreliable responses across modalities when IR contrast decreases or ToF measurements become locally corrupted. Consequently, effective drogue perception requires not only cross-modal information exchange but also mechanisms capable of selectively regulating the reliability of heterogeneous features while preserving real-time efficiency on airborne edge hardware.

In parallel, multimodal object detection has evolved from naive feature addition or concatenation toward adaptive cross-modal interaction. DAMSDet employs adaptive feature fusion and competitive query selection for multispectral detection [21]. ICAFusion introduces iterative cross-attention to exchange complementary information between modalities [22]. MMI-Det systematically investigates multimodal integration for visible–infrared object detection [23], whereas CDC-YOLOFusion employs cross-scale dynamic convolution to strengthen cross-modal feature interaction [24]. Related RGB–D studies have explored lightweight multimodal modeling from the perspectives of depth quality and speed–accuracy trade-offs [25], while LASFNet further demonstrates the effectiveness of compact attention-based fusion for resource-constrained multimodal detection [26]. These studies indicate the value of adaptive multimodal interaction. However, they are primarily designed for generic RGB–IR or RGB–D benchmarks rather than near-range IR–ToF drogue detection, where modality-specific degradation, substantial target-scale variation, and stringent airborne edge-computing constraints must be addressed simultaneously.

Despite the above progress, three issues remain insufficiently addressed. First, existing drogue perception systems predominantly rely on monocular vision, stereo vision, RGB–D sensing, or separately processed ranging measurements, whereas tightly registered IR–ToF sensing for drogue detection remains comparatively underexplored. Moreover, when infrared images suffer from low contrast or weak target boundaries, discriminative information may already be attenuated before multimodal feature extraction. Second, IR and depth features differ substantially in physical meaning, statistical distribution, and degradation characteristics. Direct addition, concatenation, or strongly coupled feature transformation can indiscriminately propagate redundant or unreliable responses, making it necessary to distinguish cross-modal interaction from reliability-aware feature selection. Third, increasing multimodal interaction complexity usually increases parameter count and computational cost, whereas practical UAV docking requires low-latency inference on resource-constrained airborne hardware.

To address these challenges, this work proposes AWIE-CGAF, a lightweight infrared–depth (IR–D) drogue detection framework designed for resource-constrained airborne perception. The framework improves multimodal representation at both the input and feature levels while maintaining deployment efficiency. At the input level, a training-free Adaptive Wavelet Image Enhancement (AWIE) module adaptively modulates structural and detail components of the infrared image in the frequency domain, thereby improving target–background discriminability without introducing an additional trainable enhancement network. At the feature level, a Cross-Gated Attention Fusion (CGAF) module separates heterogeneous cross-modal interaction from reliability-aware feature selection. Specifically, the Multimodal Feature Interaction (MFI) submodule maintains modality-specific representation pathways while exchanging complementary contextual information between the IR and depth streams, whereas the Independent Gating Unit (IGU) selectively regulates the interaction-enhanced features to suppress redundant or unreliable responses. The resulting dual-stream architecture is designed to retain heterogeneous discriminative information while satisfying real-time edge deployment requirements.

The principal contributions of this work are summarized as follows:

(1) A training-free AWIE module is developed for infrared input refinement. By adaptively modulating structural and detail information in the wavelet domain, AWIE improves the discriminability of drogue-related features without introducing additional trainable enhancement network parameters.
(2) A decoupled CGAF module is developed for heterogeneous IR–D feature fusion. MFI establishes modality-specific cross-modal interaction, while IGU performs independent reliability-aware feature regulation, thereby reducing indiscriminate feature mixing and redundant information propagation.
(3) A synchronized and pixel-wise registered Drogue Infrared–Depth (DIRD) dataset is constructed for IR–D drogue detection under four representative conditions: cloud clutter, strong glare, near-range occlusion, and normal scenes. Experiments on the DIRD dataset showed that AWIE-CGAF achieved 89.5% mAP@0.5 with 13.5 M parameters and 51.7 FPS on a Jetson AGX Orin (NVIDIA Corporation, Santa Clara, CA, USA), providing an effective balance between detection performance and real-time edge inference.

## 2. Materials and Methods

### 2.1. Overall Network Architecture

**Figure 1:**
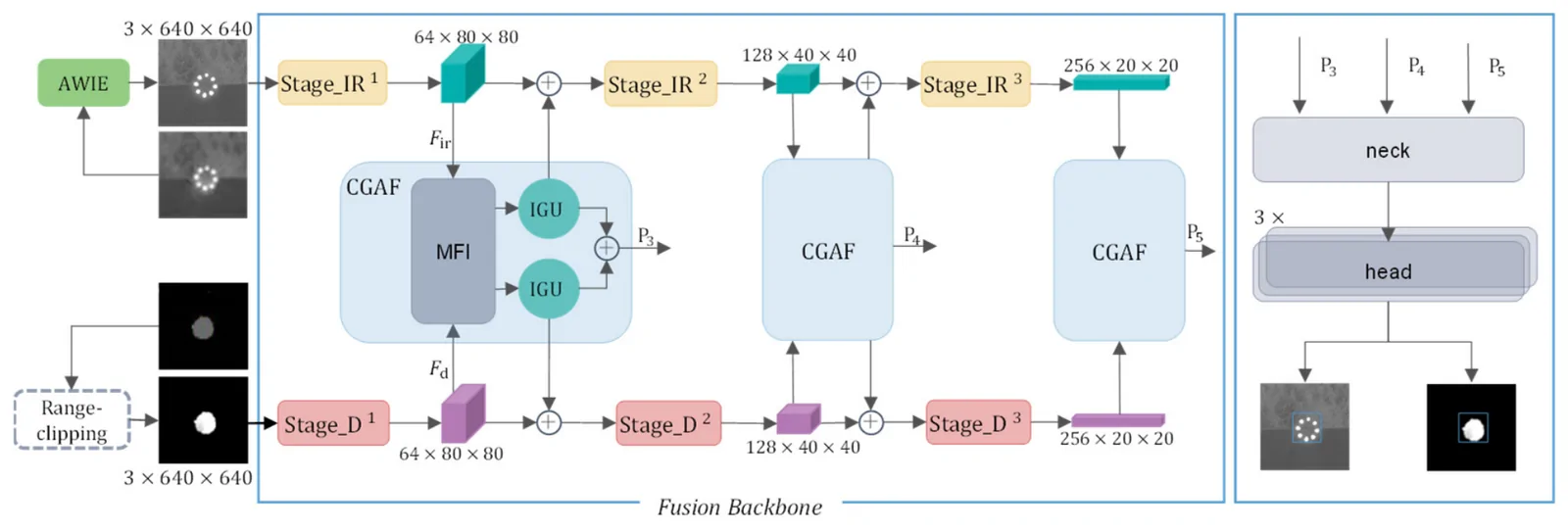
Overall architecture of the proposed AWIE-CGAF IR–D fusion-based detection framework.

The drogue detection task is formulated as a dual-modal object-detection problem using spatially registered infrared and depth images. Given pixel-level registered infrared images $I^{IR}$ and depth images $I^{D}$, the objective is to predict the category labels and bounding boxes {b,y} of the drogue target. The infrared modality characterizes the thermal radiation properties of the target, offering stability under drastic illumination changes or low-visibility conditions; the depth modality provides direct geometric and scale information that supports contour and structural discrimination. Although the two modalities are physically complementary, three key challenges persist in edge deployment scenarios: (i) representation conflicts arising from modal heterogeneity (significant distribution differences between thermal textures and geometric structures), (ii) cross-modal redundancy and noise propagation (prone to feature contamination under strong background interference), and (iii) real-time constraints due to limited resources (sensitivity to parameter count and computational power on platforms such as Jetson). To address these issues, a lightweight IR–D fusion detection framework, termed AWIE-CGAF, is developed, as illustrated in Figure 1.

The AWIE-CGAF framework comprises three main components:

(1) Dual-stream feature encoder: This employs two parameter-unshared symmetric backbone networks to extract multi-scale features $\left\{F_{l}^{IR}\right\}$ and $\left\{F_{l}^{D}\right\}$ from the infrared and depth modalities, respectively.
(2) AWIE module: Positioned at the network frontend, this performs training-free frequency-domain enhancement on the input infrared images to improve the representation quality of structural details with minimal computational overhead.
(3) CGAF module: This executes selective cross-modal fusion within the mid-to-high-level semantic feature space. This module is constructed by cascading the Multimodal Feature Interaction (MFI) submodule and the Independent Gating Unit (IGU) submodule: MFI is responsible for “decoupled interaction,” while IGU handles “reliability screening,” thereby suppressing feature aliasing and redundancy while exploiting complementarity.

### 2.2. Adaptive Wavelet-Based Image Enhancement Module

On airborne edge platforms, constrained by both computational and latency budgets, the input resolution is typically downscaled to a relatively low level. When compounded by degradation factors such as cloud scattering, glare, and noise, the edge and weak-texture responses of targets are further diluted, emerging as one of the primary bottlenecks restricting detection performance on edge platforms. Although trainable deep enhancement networks can improve visual quality, their additional parameters and computational overhead impose a significant penalty on end-to-end inference latency, which may hinder real-time deployment on resource-constrained edge platforms. Instead of employing a trainable enhancement network, AWIE performs deterministic, training-free frequency-domain modulation of the infrared input.

Deep learning enhancers typically incur substantial computational overhead; AWIE avoids this by design. While conventional Retinex-style methods [27] rely on global illumination estimation and are prone to background interference, AWIE leverages the multi-resolution and time-frequency localization properties of the wavelet transform [28]. By decomposing the image into multi-scale sub-bands, it separates structural content from local detail, applying targeted gains to each. The pipeline uses only fixed linear transforms and pointwise operations, requires no backpropagation or parameter tuning, and runs in O(N) time, meeting the real-time demands of edge platforms.

Specifically, given an input image $I\in R^{H\times W\times C}$, AWIE processes each channel independently. For each channel c, an L-level Discrete Wavelet Transform (DWT) is first performed to decompose the image into a low-frequency sub-band $A_{L}^{c}$, representing the approximate structure, and a set of high-frequency detail sub-bands $D_{l,s}^{c}$ across scales l=1,2,⋯,L. The high-frequency sub-bands contain directional detail information across three orientations: horizontal (H), vertical (V), and diagonal (D). The spatial size (side length) of each sub-band is jointly determined by the original image dimensions and the decomposition level L; as the scale l increases, the side length decreases proportionally by a factor of $1/2^{l}$. For instance, in an L-level decomposition, the side length of the low-frequency sub-band $A_{L}^{c}$ is $1/2^{L}$ of the original image.

Low-frequency sub-band enhancement: Improving global structural contrast via adaptive contrast adjustment.

(1) $${\hat{A}}_{L}^{c}=\lambda \cdot \frac{A_{L}^{c}}{\sigma \left(A_{L}^{c}\right)+\varepsilon }+\left(1-\lambda \right)\cdot A_{L}^{c},$$

Here, $\sigma (A_{L}^{c})$ denotes the global standard deviation of sub-band $A_{L}^{c}$, serving as a quantitative metric for contrast. $\varepsilon =1\times 10^{-6}$ is a numerical stability constant. λ=0.5 represents the fusion weight, determined based on the over-enhancement suppression principle outlined in Ref. [29] and the weighted fusion practice described in Ref. [30]. The normalization factor is defined as:

(2) $$k_{l}^{c}=\frac{1}{\sigma (A_{L}^{c})+\varepsilon },$$

This mechanism enables the enhancement magnitude to adaptively respond to image contrast: for low-contrast images, the smaller $\sigma (A_{L}^{c})$ results in a larger enhancement factor $k_{l}^{c}$, thereby increasing the enhancement magnitude; the opposite applies to high-contrast images. Consequently, adaptive contrast stretching is realized with fixed parameters.

High-frequency sub-band enhancement: A unified enhancement function is applied to the high-frequency sub-band in the horizontal (H), vertical (V), and diagonal (D) directions:

(3) $${\hat{D}}_{l,s}^{c}=D_{l,s}^{c}\cdot \left(1+\beta \cdot \gamma_{l}\right),$$

Here, $D_{l,s}^{c}$ and ${\hat{D}}_{l,s}^{c}$ denote the original and enhanced coefficients, respectively, at scale l, orientation s (s∈{H,V,D}), and channel c. β is a nonlinear enhancement factor fixed at 0.8 to regulate the high-frequency detail enhancement magnitude. $\gamma_{l}$ represents the scale-wise enhancement weight, satisfying $0<\gamma_{l}<1$. The scaling factor $\left(1+\beta \cdot \gamma_{l}\right)$ is confined to the open interval (1, 1.8) to suppress noise and artifacts. The enhancement weight $\gamma_{l}$ is adaptively computed based on the normalized average energy of all high-frequency directional sub-bands at this scale:

(4) $$\gamma_{l}=\frac{1}{{\bar{E}}_{l}+\varepsilon },{\bar{E}}_{l}=\frac{1}{K}\sum_{s=1}^{K}\frac{{\left.\left\|D_{l,s}^{c}\right.\right\|}_{F}^{2}}{{\left.\left\|D_{l,s}^{c}\right.\right\|}_{F}^{2}+\delta },$$

Here, $D_{l,s}^{c}$ denotes the original high-frequency sub-band coefficient matrix, ${\left\|\cdot \right\|}_{F}$ represents the Frobenius norm, K=3 is the total number of orientations, and $\varepsilon =\delta =1\times 10^{-6}$ are numerical stability constants. This design is grounded in the physical characteristics of low-contrast imaging: edge and texture information of the drogue manifests as high-frequency components with significantly attenuated energy in the wavelet domain. When the normalized average energy ${\bar{E}}_{l}$ at scale l is low, $\gamma_{l}$ increases, prompting the algorithm to enhance high-frequency details across all orientations at this scale, thereby improving weak feature responses pertinent to the detection task.

Finally, the enhanced image $\hat{I}$ is reconstructed via the inverse discrete wavelet transform (IDWT) using the enhanced low-frequency approximation coefficients and high-frequency detail coefficients. The entire pipeline exclusively comprises DWT/IDWT and pointwise operations, eliminating the need for backpropagation or parameter updating. Consequently, the proposed method offers distinct advantages in terms of stability, controllability, and computational efficiency.

**Figure 2:**
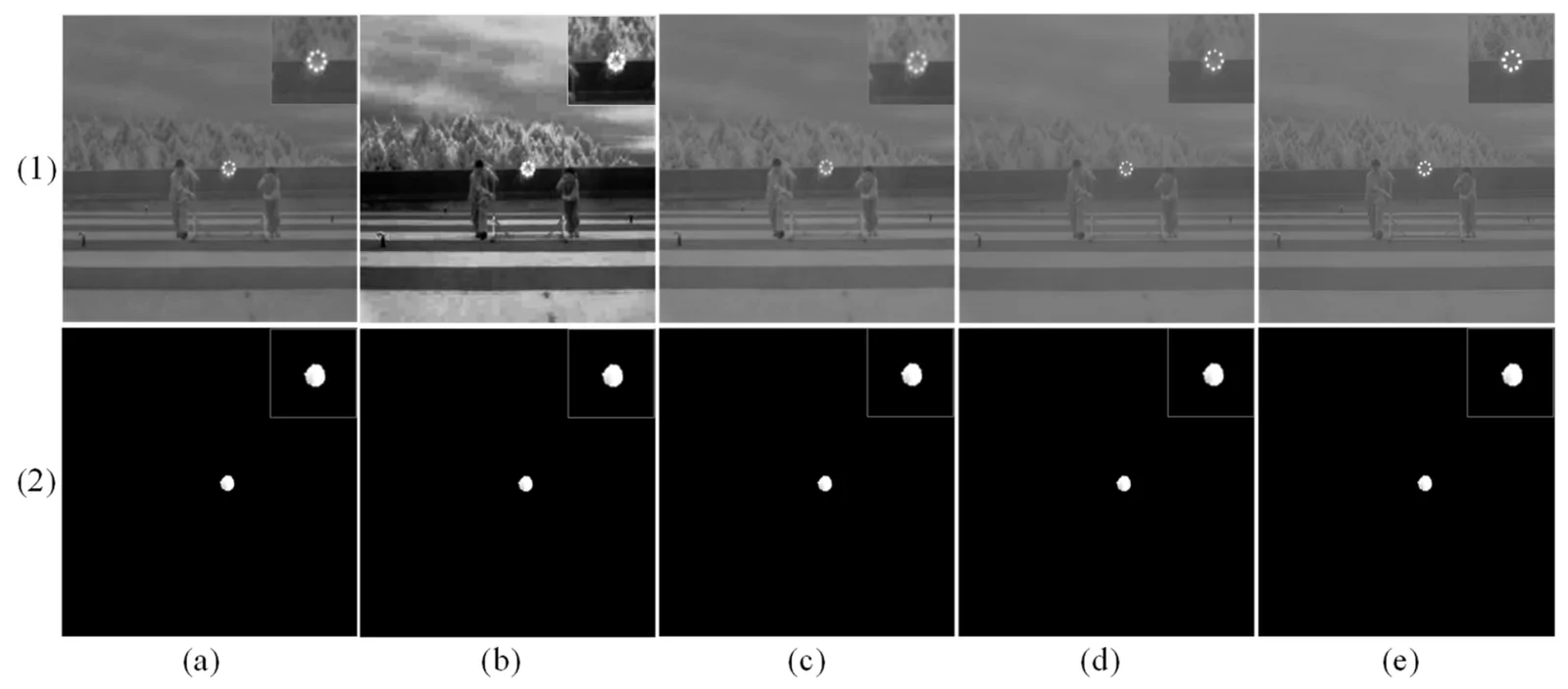
Qualitative comparison of different infrared preprocessing methods on the drogue detection task. Rows correspond to (**1**) an infrared image, (**2**) a depth image; columns correspond to (**a**) original image, (**b**) GHE, (**c**) CLAHE, (**d**) MSR, and (**e**) proposed AWIE.

To quantify the benefit of the proposed AWIE for IR–D detection, comparative experiments were conducted on the DIRD dataset. As shown in Figure 2, the preprocessing stage consists of two independent branches: (1) infrared preprocessing, where an enhancement algorithm is applied to improve IR image quality; and (2) depth preprocessing, where only a range clipping operation is used for data cleaning, without any enhancement. In all experiments, depth preprocessing was fixed to range clipping within 0.3–10 m, while only the IR enhancement method was altered to eliminate confounding factors. The compared IR schemes include: (a) no enhancement, (b) Global Histogram Equalization (GHE), (c) Contrast-Limited Adaptive Histogram Equalization (CLAHE) [31], (d) Multi-Scale Retinex (MSR) [32], and (e) the proposed AWIE. All experiments employed identical detection architectures and training protocols to ensure a fair comparison. mAP@0.5 on the test set served as the primary task metric. Complementary full-reference quality was evaluated using peak signal-to-noise ratio (PSNR) [33] and structural similarity index measure (SSIM) [34]. PSNR and SSIM employed the unprocessed original infrared image as the reference standard, against which all enhanced outputs were compared. Table 1 marks the “Original image” row with “–”, as this anchoring reference is exempt from comparative evaluation.

Runtime was benchmarked on an NVIDIA Jetson AGX Orin in MAX-N mode with pipelined IR and depth preprocessing. Table 1 lists the per-frame IR preprocessing latency averaged across ten independent runs. Depth range clipping held steady at 0.32 ms and contributed little to the total; thus, the overall preprocessing cost was dominated by the IR branch. All compared methods remained below 5 ms in end-to-end latency, meeting the real-time constraint for airborne edge deployment.

Table 1 shows that AWIE struck a better balance among fidelity, detection accuracy, and compute cost than competing methods. AWIE reached the highest PSNR/SSIM at 34.9 dB/0.993, preserving edges and coarse structure while holding measurement noise down. The IR preprocessing step took 2.2 ms, just 0.5 ms longer than the fastest CLAHE at 1.7 ms, yielding an 8.6 dB PSNR improvement and a 0.014 SSIM gain. Feeding AWIE-processed IR to the baseline detector pushed mAP@0.5 to 85.3%, a 1.5-point increase over raw inputs. Visual comparisons in Figure 2 show that GHE often over-exposed and distorted structure, CLAHE introduced block artifacts near boundaries, and MSR tended to produce halos with uneven local contrast. In contrast, AWIE resulted in sharper, more consistent edge-texture responses on the drogue and suppressed noise across homogeneous background regions, delivering cleaner input to downstream cross-modal fusion.

### 2.3. Cross-Gated Attention Fusion Module

Infrared and depth features are fused through CGAF, which consists of an MFI submodule followed by an IGU submodule. Its architecture adopts a decoupled interaction–independent selection strategy, keeping modality-specific features intact while performing efficient cross-modal fusion.

(1) MFI submodule: decoupled cross-modal interaction

**Figure 3:**
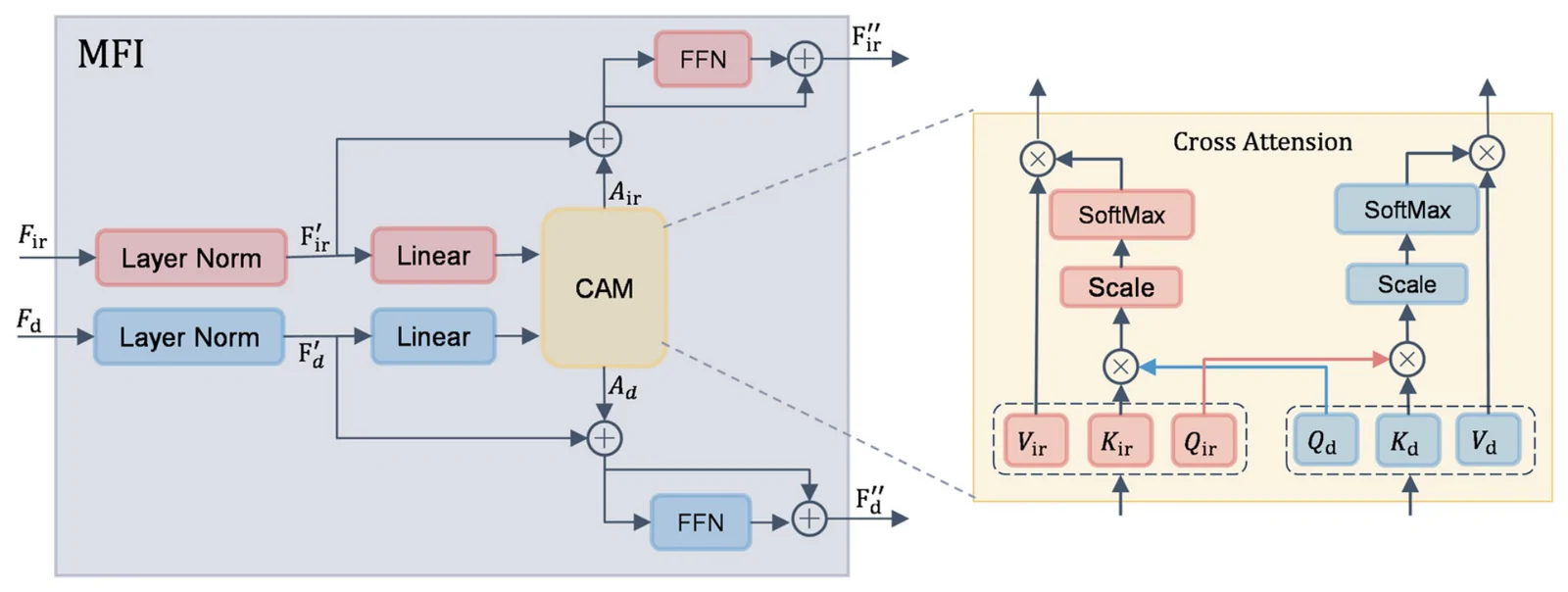
Architecture of the proposed MFI (Modality Feature Interaction) module.

Figure 3 shows the MFI submodule. It uses a dual-path decoupled cross-attention mechanism to alleviate representation conflicts when heterogeneous modalities are projected into a shared space. Infrared features primarily encode thermal radiation cues, while depth features emphasize geometric structure information. Applying shared projection parameters compresses heterogeneous distributions into a common latent space and weakens discriminative modality-specific representations.

To address this issue, MFI assigns independent Query (*Q*), Key (*K*), and Value (*V*) projection matrices to each modality. Given spatially aligned infrared and depth features $F_{ir}$ and $F_{d}$, layer normalization is first applied to obtain $F_{ir}^{\prime }$ and $F_{d}^{\prime }$. The modality-specific *Q*–*K*–*V* representations are then generated as:

(5) $$Q_{ir},K_{ir},V_{ir}=F_{ir}^{\prime }W_{ir}^{Q},F_{ir}^{\prime }W_{ir}^{K},F_{ir}^{\prime }W_{ir}^{V},$$

(6) $$Q_{d},K_{d},V_{d}=F_{d}^{\prime }W_{d}^{Q},F_{d}^{\prime }W_{d}^{K},F_{d}^{\prime }W_{d}^{V}$$

During cross-modal interaction, the infrared branch uses its query vector $Q_{ir}$ to retrieve complementary contextual information from the depth modality via ($K_{d},V_{d}$). To enhance spatial structure modeling, a lightweight relative positional bias, denoted as B, is incorporated into the attention logits [35], whose parameter size is independent of the input resolution:

(7) $${\mathrm{Attn}}_{ir}=\mathrm{softmax}\left(\frac{Q_{\mathrm{ir}}K_{d}^{T}}{\sqrt{d_{k}}}+B\right)V_{d},$$

(8) $$\left.F_{ir}^{\prime \prime }{=(\mathrm{Attn}}_{ir}+F_{ir}^{\prime }{)+\mathrm{FFN}(\mathrm{Attn}}_{ir}+\;F_{ir}^{\prime }\right),$$

where $d_{k}$ denotes the key dimension and FFN(⋅) represents a feed-forward network (FFN). The enhanced depth feature $F_{d}^{\prime \prime }$ is obtained symmetrically. By decoupling the projection paths, MFI effectively avoids representation contamination caused by shared transformations, enabling the enhanced features to simultaneously preserve modality-specific discriminative cues and complementary cross-modal context.

(2) IGU submodule: independent gating and feature selection

**Figure 4:**
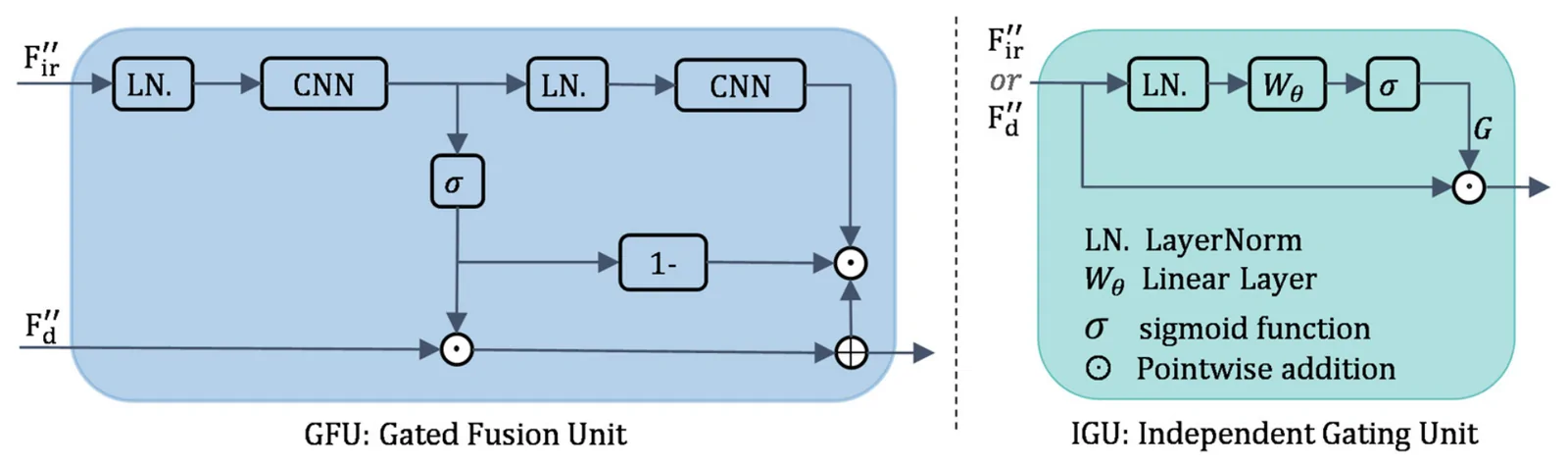
Structural comparison between the GFU and the proposed IGU.

To further select informative representations from the interaction-enhanced features, a lightweight IGU is cascaded after MFI, as shown in Figure 4. Unlike conventional shared gating mechanisms, e.g., the Gated Fusion Unit (GFU) [36], which employs cascaded 1×1 convolutions for feature filtering, IGU adopts two parallel independent parameter paths for different modalities, thereby avoiding premature feature entanglement before gating. For the infrared branch, the enhanced feature $F_{ir}^{\prime \prime }$ is first normalized and then projected through a modality-specific linear transformation to generate a content-adaptive gating mask:

(9) $$G_{ir}=\sigma \left(W_{\theta }^{ir}\cdot \mathrm{LN}\left(F_{ir}^{\prime \prime }\right)\right),$$

where σ(⋅) denotes the sigmoid activation function and $W_{\theta }^{ir}\in [0,1]$ represents the dynamic gating weights. The feature is subsequently modulated via element-wise multiplication:

(10) $$F_{ir}^{\mathrm{out}}=F_{ir}^{\prime \prime }⊙G_{ir},$$

where ⊙ denotes element-wise multiplication. The depth branch output $F_{d}^{out}$ is obtained analogously. Finally, the fused multimodal representation is generated through element-wise addition:

(11) $$F_{\mathrm{fuse}}^{\mathrm{out}}=F_{ir}^{\mathrm{out}}+F_{d}^{\mathrm{out}},$$

The IGU design targets edge deployment, with efficiency and discriminability both taken into account. Its parallel structure removes inter-layer serial dependency and aids hardware acceleration. Independent parameter paths keep modality-specific discriminative information intact and amplify it where needed. The resulting fused feature $F_{fuse}^{out}$ is then fed into both the backbone and neck networks to enrich cross-modal context and support downstream object detection.

## 3. Results and Discussion

### 3.1. Experimental Setup

Experiments included offline training and edge deployment. Training was conducted on a single NVIDIA RTX 4090 GPU with twenty-four gigabytes of memory utilizing PyTorch 2.0.1 and CUDA 11.8. Deployment efficiency was tested on an NVIDIA Jetson AGX Orin platform equipped with sixty-four gigabytes of LPDDR5 memory and running JetPack 5.1.2 in MAX-N performance mode. All software dependencies and library versions were fixed to ensure reproducibility.

Performance was evaluated using accuracy, model complexity, and inference efficiency. Following the COCO 2017 protocol [37], detection results are reported as mAP@0.5 and mAP@0.5:0.95. Localization accuracy was evaluated using the Center Location Error (CLE), calculated as the Euclidean distance between predicted and ground-truth bounding box centers on the original 640 × 640 input resolution. For latency measurement, models were converted to TensorRT 8.5 under FP16; FPS values, averaged over ten independent inference runs, were measured on the target edge platform. Training followed the standard YOLOv8 training recipe. Training employed stochastic gradient descent for 200 epochs with a batch size of 16, an initial learning rate of 0.01 with a cosine annealing learning rate schedule, a momentum of 0.937, and a weight decay of 5 × 10^−4^.

AWIE-CGAF was compared with representative methods. These included YOLOv8n on infrared-only and depth-only data, and several fusion methods such as LASFNet [26], GAFF [38], LRAF-Net [39], GFD-SSD [40], ICAFusion [22], CDC-YOLOFusion [24], CFT [41], and DAMSDet [21].

### 3.2. Dataset

To evaluate the proposed AWIE-CGAF under complex degradations, we constructed an IR–D dataset termed DIRD for drogue detection. DIRD consists of strictly synchronized and pixel-wise registered IR images and ToF depth maps, covering representative challenging conditions, such as cloud clutter, strong glare, and near-range occlusions.

#### 3.2.1. Data Acquisition and Registration

**Figure 5:**
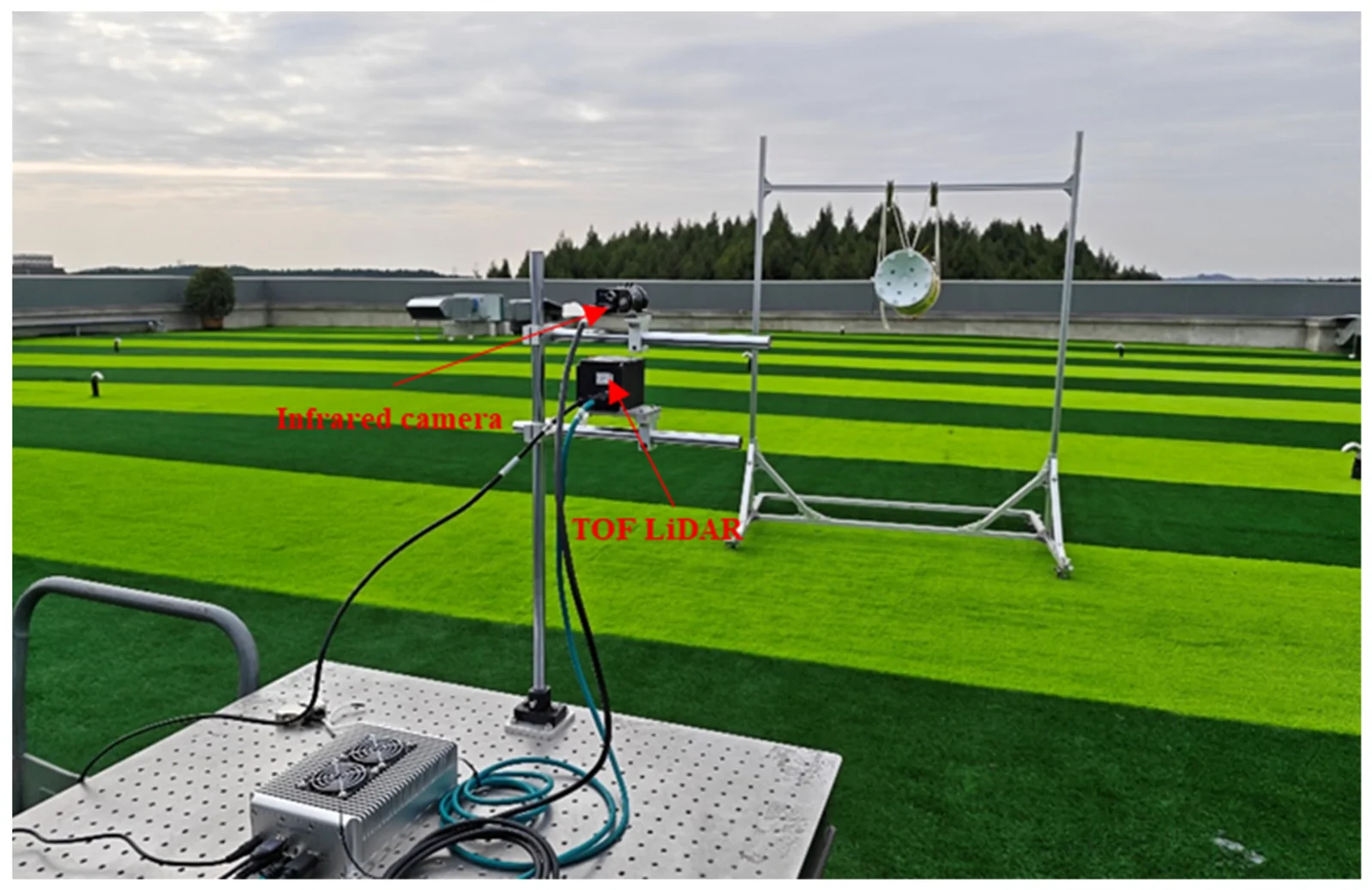
Photograph of the deployed IR–D data acquisition platform in the field.

As shown in Figure 5, the acquisition platform comprised an infrared camera and a ToF imaging LiDAR rigidly mounted with hardware triggering for strict synchronization (50 Hz). Pixel-level alignment was obtained via the following registration procedure: (1) each sensor was calibrated using Zhang’s method to estimate intrinsics and distortion parameters [42]; (2) the extrinsic parameters between sensors (rotation R and translation t) were estimated using a calibration board [43]; (3) depth maps were projected into the IR camera coordinate system and mapped onto the IR image plane to generate registered IR–D pairs.

#### 3.2.2. Annotation Protocol and Scale Statistics

DIRD contains 5116 registered IR–D pairs with a fixed resolution of 640×640. Annotations follow a normalized format, where each record includes the class index, normalized box center (x,y), and normalized width/height (w,h) in [0,1]. DIRD is a single-class detection dataset, and each image pair contains exactly one drogue instance, resulting in 5116 annotations in total, ensuring consistent labeling and controlled statistics. To characterize target scales, we computed the ratio of bounding box area to image area and categorized instances into small (<5%), medium (5–15%), and large (>15%) targets. The overall scale distribution comprised 27%, 45%, and 28% for small, medium, and large targets, respectively, covering the scale variation from long-range approach to near-range docking. Dedicated characterization of drogue attitude was not performed in the current ground-based experiments, with quantitative evaluation reserved for future wind tunnel and flight tests.

#### 3.2.3. Challenging Conditions and Data Split

**Figure 6:**
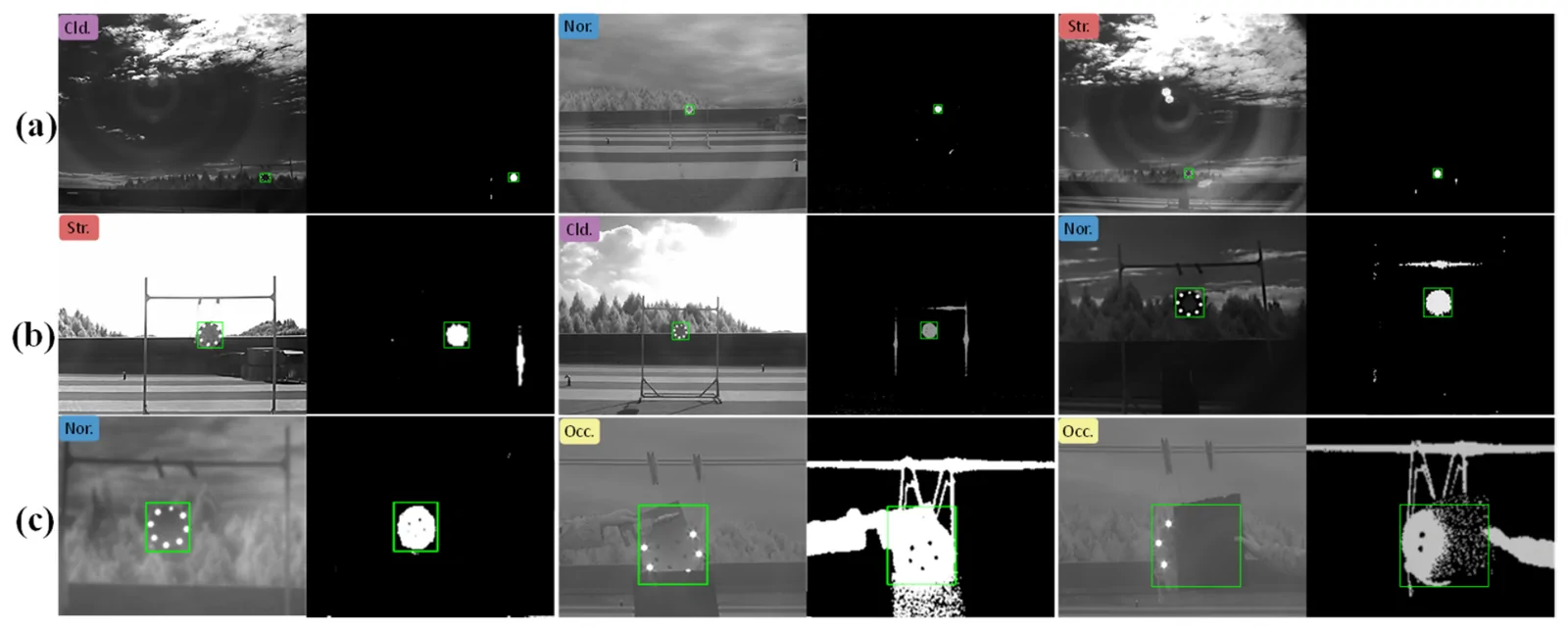
Representative DIRD samples. Rows correspond to (**a**) small-scale targets, (**b**) medium-scale targets, and (**c**) large-scale targets; columns present registered IR–D pairs. Top-left color codes indicate scene types: normal (Nor.), cloud clutter (Cld.), strong glare (Str.), and near-range occlusion (Occ.). The green bounding boxes indicate ground-truth drogue locations.

Figure 6 illustrates representative DIRD dataset samples, each comprising a spatially registered IR–D image pair, associated ground-truth bounding boxes, and top-left color-coded marker denoting the corresponding environmental condition. The samples span the full range of target scales and challenging UAV docking scene types examined in this work, with statistical distributions tabulated in Table 2.

DIRD contains four scene types: cloud clutter (Cld.), strong glare (Str.), near-range occlusion (Occ.), and normal (Nor.). Each independent acquisition sequence corresponds to a single, fixed scene type. The dataset was therefore partitioned strictly at the sequence leveland split at a 7:2:1 ratio into train/val/test sets, yielding 3581, 1023, and 512 image pairs. Since each subset consists of complete sequences, the proportions of the four scene types and target scale distributions remain nearly identical across subsets (Table 2).

### 3.3. Comparative Analysis

To evaluate the performance upper bound of different fusion strategies under high-quality inputs, all comparative experiments employed AWIE as a unified preprocessing module. Since the effectiveness of AWIE was already validated in Section 2.2, this section focuses on analyzing the fusion capability of CGAF itself. The independent contribution of AWIE is further quantified in ablation studies in Section 3.4.

#### 3.3.1. Quantitative Comparison with Competing Methods

All methods were evaluated on the DIRD dataset with AWIE-enhanced IR–D inputs. Compared models spanned lightweight fusion networks, attention-based fusion approaches, visible–infrared fusion frameworks adapted to the IR–D setting, Transformer-based methods, single-modal detectors, and the baseline model.

The baseline adopted a dual-stream architecture with YOLOv8n backbones integrated with a simple additive fusion strategy. Specifically, infrared and depth inputs were processed by two independent streams, each built upon a YOLOv8n backbone. At the P3–P5 feature levels, corresponding feature maps underwent element-wise addition to integrate information. This dual-stream additive-fusion model served as the benchmark. Consistent with the training protocol in Section 3.1, the baseline and all comparative methods share identical training hyperparameters. Quantitative results are given in Table 3.

Results show that AWIE-CGAF achieved the best overall balance among detection accuracy, model complexity, and deployment efficiency.

(1) Detection accuracy: AWIE-CGAF reached 89.5% mAP@0.5 and 58.2% mAP@0.5:0.95, giving the best overall result. Compared with LASFNet at 13.9 M parameters, mAP@0.5 rose by 4.8 percentage points. Against DAMSDet at 120.5 M parameters, the increase was 5.2 points. These gaps suggest that the fusion design achieved accuracy improvements without relying on a substantial increase in model capacity.
(2) Model efficiency: AWIE-CGAF kept complexity low with 13.5 M parameters and 25.6 GFLOPs while achieving the highest accuracy. It requires just 19.9% of the parameters and 36.3% of the computational cost compared with CDC-YOLOFusion, which reached comparable performance, demonstrating a superior accuracy–efficiency trade-off.
(3) Edge deployment performance: On the NVIDIA Jetson AGX Orin platform, AWIE-CGAF achieved a real-time inference speed of 51.7 FPS, which meets the typical real-time throughput (~50 FPS) expected for UAV onboard vision systems [44,45] while outperforming CDC-YOLOFusion by 27.7% in deployment speed.
(4) Localization accuracy: Quantitative evaluation on the test set yielded a mean CLE of 3.2 pixels for AWIE-CGAF. Relative to the 6.8 pixels baseline, the proposed method reduced the mean CLE by 52.9%, indicating improved center localization on the DIRD test set.

#### 3.3.2. Qualitative Comparison with Competing Methods

**Figure 7:**
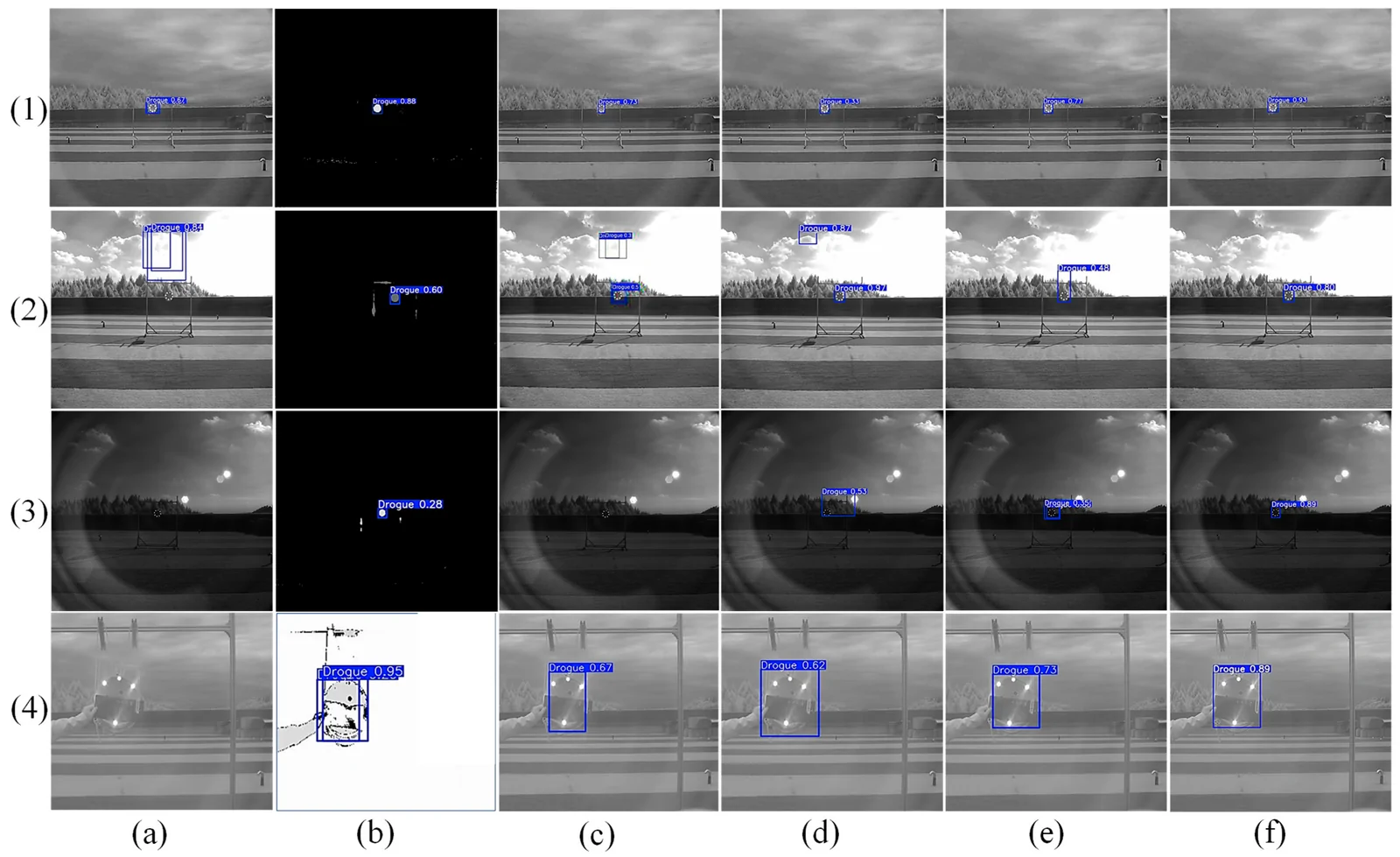
Qualitative comparison of detection algorithms under different interference scenarios. Rows correspond to (**1**) normal (Nor.), (**2**) cloud clutter, (**3**) strong glare, (**4**) near-range occlusion; columns correspond to (**a**) single-modality infrared detection, (**b**) single-modality depth detection, (**c**) multimodal baseline, (**d**) ICAFusion, (**e**) CDC-YOLOFusion, (**f**) AWIE-CGAF.

To further examine the behavior of CGAF under challenging conditions, its qualitative performance was compared to ICAFusion and CDC-YOLOFusion, the top-performing baselines according to Table 3. Qualitative comparisons are shown in Figure 7.

Under normal conditions, single-modality infrared detection (a) produced two overlapping bounding boxes for the same target instance; the multimodal baseline (c) yielded undersized bounding boxes; single-modality depth detection (b), ICAFusion (d), and CDC-YOLOFusion (e) yielded correct detections, yet with lower confidence scores than AWIE-CGAF (f).

Under cloud clutter, single-modality infrared detection (a), the multimodal baseline (c), and ICAFusion (d) falsely detected cloud masses as targets; single-modality depth detection (b) produced correct detections, albeit with lower confidence than AWIE-CGAF (f); CDC-YOLOFusion (e) yielded correct detections, but with loose bounding boxes.

Under strong glare, single-modality infrared detection (a) and the multimodal baseline (c) missed the target; single-modality depth detection (b) produced correct detections, albeit with lower confidence than AWIE-CGAF (f); ICAFusion (d) and CDC-YOLOFusion (e) yielded correct detections, but with loose bounding boxes.

Under near-range occlusion, single-modality infrared detection (a) missed the target; single-modality depth detection (b) output two overlapping bounding boxes for the same instance; the multimodal baseline (c) yielded undersized bounding boxes; ICAFusion (d) and CDC-YOLOFusion (e) yielded correct detections, yet with lower confidence scores than AWIE-CGAF (f).

The following discussion synthesizes the quantitative results from Table 3 with the qualitative findings from Figure 7. LASFNet (13.9 M) applies post-fusion global attention for global context modeling; the early fusion might be related to homogenized dilution of modality-specific representations. CGAF comprised 13.5 M parameters and preserved independent representation paths in the feature interaction phase, leveraging the IGU to filter complementary features. Under a comparable parameter budget, it alleviated feature entanglement and improved mAP@0.5 by 4.8 percentage points to 89.5%. ICAFusion resorted to iterative cross-attention, incurring 25.9 M parameters and 47.6 GFLOPs of computational cost. By contrast, AWIE-CGAF adopts a decoupled design that disentangles alignment from reliability estimation to eliminate redundant attention computations. It constrained high-cost interactions to high-response regions, reducing computation by approximately 46.2%, and increasing mAP@0.5 by 3.6 percentage points. CDC-YOLOFusion employs cross-scale dynamic convolution to reinforce local context aggregation at high computational cost. CGAF reduced computation by approximately 64% relative to CDC-YOLOFusion, requiring 25.6 GFLOPs, and replaced dense local convolution with global interaction. The IGU adaptively modulated semantic features based on reliability information, achieving complementary feature fusion while preserving independent representation paths for IR and Depth. This resulted in a balanced optimization of accuracy and efficiency.

These quantitative advantages are corroborated by the qualitative results in Figure 7, where CGAF maintained correct target detections in the representative challenging cases shown in Figure 7. In the presence of modality degradation, single-modality methods lack complementary information, predisposing such methods to false alarms, missed detections, and bounding box overlap. The naive additive fusion strategy fails to discriminate degraded features; its indiscriminate summation dilutes edge responses, prompting regression box shrinkage below the ground-truth scales; the absence of reliability screening incurs missed detections and response inconsistencies. ICAFusion’s iterative cross-attention itself, despite emphasizing channel-wise correlations, imposes no explicit spatial constraints, e.g., relative position bias, leaving contours devoid of tailored regularization. The result is that under interference, bounding box tightness erodes. CDC-YOLOFusion is constrained by its limited local receptive field, hindering the acquisition of global geometric priors and making it prone to incorporating neighboring backgrounds into predicted boxes. Under strong glare, local convolutions tend to misidentify aliased textures within highlight-saturated regions as valid structural edges, further inducing loose boundary regression and reduced localization compactness, a geometric degradation driven by local inductive biases that becomes particularly pronounced in cluttered and glaring scenarios. CGAF incorporates cross-attention with relative position bias, suppressing the boundary relaxation observed in ICAFusion. By enabling global cross-attention interactions that transcend local convolutional neighborhoods, it mitigates the receptive-field limitations inherent in CDC-YOLOFusion. Furthermore, an independent gating mechanism decouples alignment from reliability estimation, alleviating feature entanglement and adaptively suppressing degraded cues.

### 3.4. Ablation Study

#### 3.4.1. Quantitative Analysis of Ablation Results

To quantify the respective contributions of AWIE and CGAF, Table 4 summarizes ablation results obtained by incrementally adding individual components.

Table 4 reports 83.8% mAP@0.5 for the baseline model. Equipping the baseline with CGAF increased parameters from 6.5 M to 13.5 M, raised mAP@0.5 to 87.5%, and registered 56.8% mAP@0.5:0.95. AWIE alone raised mAP@0.5 to 85.3%. With AWIE and CGAF, mAP@0.5 advanced to 89.5%, a 5.7 percentage point gain over the baseline; mAP@0.5:0.95 climbed from 49.6% to 58.2%.

A further component-wise ablation was conducted for MFI and IGU, as reported in Table 5. Introducing only MFI improved mAP@0.5 from 83.8% to 84.8%. Integrating IGU yielded the full CGAF, culminating in 87.5% mAP@0.5 and 56.8% mAP@0.5:0.95.

#### 3.4.2. Qualitative Analysis of Ablation Results

**Figure 8:**
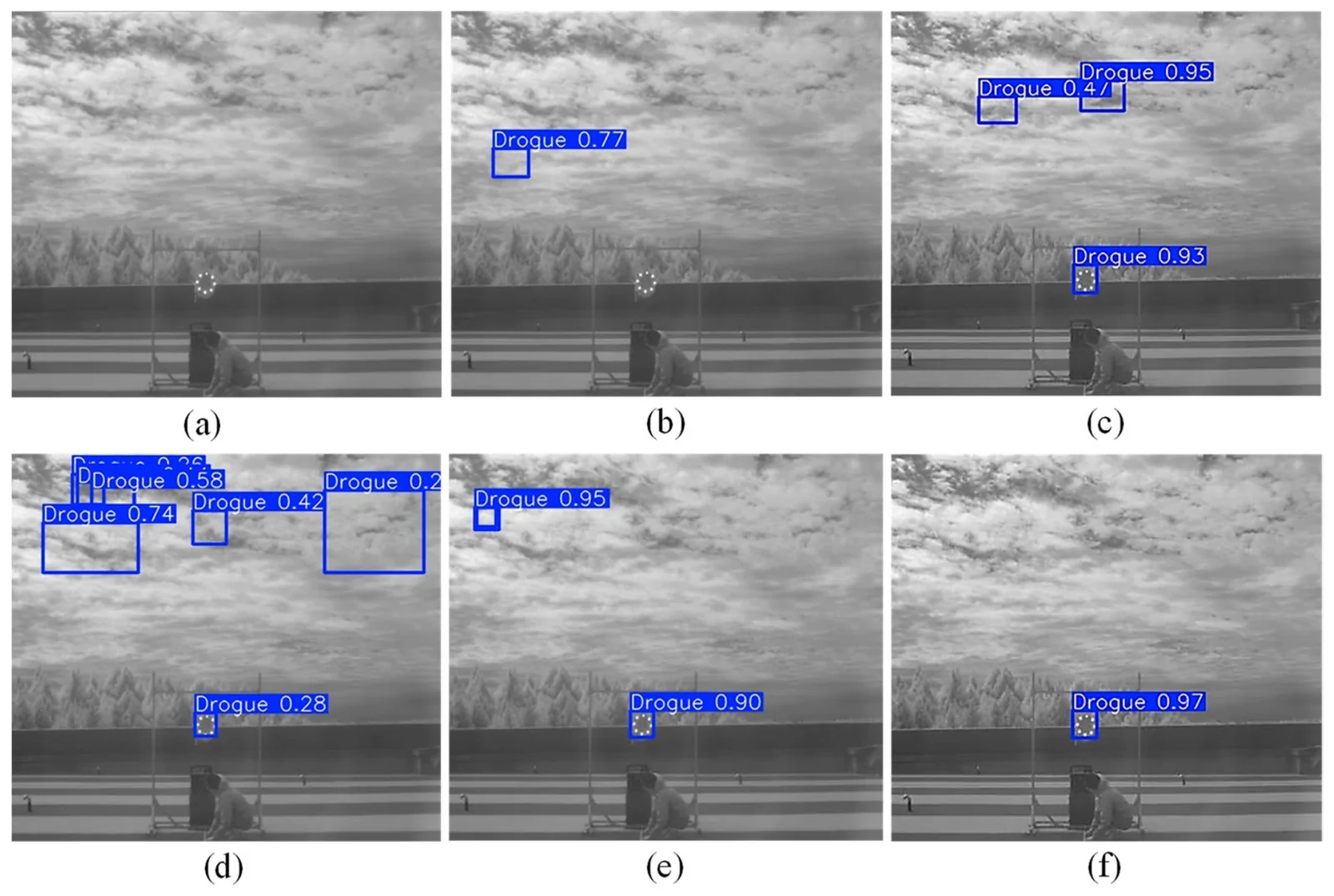
Qualitative comparison of ablation experiment results. (**a**) Original image; (**b**) baseline model; (**c**) baseline + AWIE preprocessing; (**d**) baseline + MFI module; (**e**) baseline + MFI + IGU (**f**) full pipeline (AWIE-CGAF).

Figure 8 used a typical cloud-clutter scene as a hard example for qualitative ablation. Semi-transparent clouds scattered ToF signals, producing localized distortions in the depth map that fragmented the drogue’s geometric structure. Clouds also emitted infrared signatures similar to the drogue, lowering target–background separability. Because the depth map no longer supplied reliable geometric constraints, it was omitted from the visualization; only IR response maps were shown to analyze how each module affected separability.

Under this scenario, the addition-based fusion baseline exhibited missed drogue detections and background false positives. Standalone AWIE preprocessing enabled successful target detection in this example, although one additional false positive was introduced. MFI recovered drogue detection but introduced redundant background activations. Cascading IGU subsequently reduced the false positive rate substantially. Following full integration of AWIE for input refinement, the complete model ultimately yielded accurate drogue localization.

Quantitative results (Table 4) indicate that AWIE-CGAF elevated mAP@0.5 to 89.5%, a 5.7 percentage point gain over the multimodal baseline. Standalone AWIE refined the frequency-domain representation and improved target detectability, but one additional false positive was observed. This behavior may be attributable to enhancement of high-frequency background-edge responses together with the desired drogue-related details.

Component-wise ablation results in Table 5 show that MFI alone increased mAP@0.5 from 83.8% to 84.8%. When IGU was subsequently added after MFI, the full CGAF configuration reached 87.5% mAP@0.5, corresponding to a further 2.7 percentage-point improvement. The qualitative results in Figure 8 indicate that MFI recovered drogue responses through cross-modal interaction but might simultaneously retain non-discriminative background activations. The cascaded IGU then suppressed these unreliable responses through adaptive gating, resulting in fewer false positives and improved feature discriminability. Therefore, MFI and IGU performed different but sequentially connected functions: MFI established complementary cross-modal interaction, whereas IGU regulated the inter-action-enhanced representations before final fusion. The 2.7 percentage-point improvement was interpreted as the incremental gain obtained by adding IGU after MFI in the current cascade.

AWIE optimized input features to enhance target–background separability, alleviating the fitting burden of subsequent modules and providing a high signal-to-noise ratio input for MFI alignment; precise MFI alignment enabled the IGU gating mechanism to filter redundant features and reinforce discriminative features. The cascade of AWIE input refinement, MFI alignment, and IGU gating ultimately yielded robust drogue localization.

## 4. Conclusions

This work introduces AWIE-CGAF, a lightweight IR–D fusion detection framework explicitly designed to address modality heterogeneity and feature redundancy while adhering to stringent real-time constraints on airborne edge platforms. The coupling of a training-free AWIE module with a CGAF architecture enables high-fidelity, temporally consistent IR–D detection on resource-constrained edge platforms. Experimental results show that the AWIE module effectively improves the input image signal-to-noise ratio and structural separability while maintaining computational efficiency. Meanwhile, the CGAF module performs adaptive cross-modal fusion across multi-scale features, preserving modality-specific information and dynamically selecting complementary features. This mechanism mitigates feature entanglement. On the DIRD dataset, an mAP@0.5 of 89.5% was achieved with 13.5 M parameters. Inference on a Jetson AGX Orin sustained 51.7 FPS, surpassing existing fusion counterparts listed in Table 3 under edge computing constraints.

Validation was performed on a stationary experimental prototype. The current setup cannot fully reproduce the aerodynamic turbulence inherent to free-flight UAV docking and therefore fails to adequately capture the resultant pronounced drogue pose variations. Constrained by ground testing conditions, the precise robustness boundaries of the proposed algorithm remain to be quantified. Wake-induced pose oscillations degrade imaging quality, manifesting as motion blur and sensor jitter. Transitioning to airborne deployment necessitates rigorous dynamic validation. Future work prioritizes large-scale wind tunnel tests and hardware-in-the-loop simulations to better approximate real-world UAV docking procedure.

## Figures and Tables

**Table 1:** Quantitative comparison of infrared preprocessing methods on the DIRD dataset.

IR Preprocessing Method	PSNR (dB)	SSIM	Preprocess Time (ms)	mAP@0.5 (%)
Original image	–	–	–	83.8
GHE	12.9	0.682	6.2	52.4
CLAHE	26.3	0.979	1.7	81.5
MSR	30.8	0.992	3.9	84.6
Proposed AWIE	34.9	0.993	2.2	85.3

**Table 2:** Scene and scale distributions of the partitioned subsets in the DIRD dataset.

Subset	Pairs	Scene Composition (%)				Scale Distribution (%)		
		Cld.	Str.	Occ.	Nor.	S	M	L
Full	5116	30	25	15	30	27	45	28
Train	3581	30	25	15	30	27	45	28
Val	1023	29	26	16	29	27	45	28
Test	512	31	24	14	31	26	45	29

**Table 3:** Performance comparison of different methods on the DIRD dataset.

Method	Modality	Params. (M)	mAP@0.5 (%)	mAP@0.5:0.95 (%)	CLE (Pixels)	FPS	GFLOPs
YOLOv8n	IR	2.6	70.1	31.2	12.7	76.5	6.4
	D	2.6	81.6	48.1	10.1	75.9	6.4
LASFNet	IR + D	13.9	84.7	54.1	4.8	52.9	29.7
GAFF	IR + D	20.5	84.1	50.4	5.6	47.9	123.3
LRAF-Net	IR + D	18.8	81.0	46.8	7.9	50.6	30.5
GFD-SSD	IR + D	25.6	79.2	43.3	9.4	49.4	54.9
ICAFusion	IR + D	25.9	85.9	56.5	5.3	47.1	47.6
CDC-YOLOFusion	IR + D	67.7	87.0	57.4	4.7	40.5	70.5
CFT	IR + D	79.2	83.9	48.8	6.3	32.4	156.1
DAMSDet	IR + D	120.5	84.3	52.1	5.1	27.5	123.3
Baseline	IR + D	6.5	83.8	49.6	6.8	67.2	16.5
AWIE-CGAF (Ours)	IR + D	13.5	89.5	58.2	3.2	51.7	25.6

**Table 4:** Ablation results of the proposed AWIE-CGAF architecture on the DIRD dataset.

Method	Params. (M)	FPS	GFLOPs	mAP@0.5 (%)	mAP@0.5:0.95 (%)
Baseline	6.5	67.2	16.5	83.8	49.6
Baseline + CGAF	13.5	56.5	25.5	87.5	56.8
Baseline + AWIE	6.5	58.9	16.6	85.3	52.3
Baseline + CGAF + AWIE	13.5	51.7	25.6	89.5	58.2

**Table 5:** Ablation results of the CGAF module.

Method	Params. (M)	FPS	GFLOPs	mAP@0.5 (%)	mAP@0.5:0.95 (%)
Baseline	6.5	67.2	16.5	83.8	49.6
Baseline + MFI	9.8	61.3	21.9	84.8	52.4
Baseline + CGAF	13.5	56.5	25.5	87.5	56.8

## Data Availability

The DIRD dataset is temporarily inaccessible due to data governance policies enforced by the China Aerodynamics Research and Development Center (CARDC). Figure 6 presents representative samples covering all predefined target scales and scene types. Detailed documentation regarding the dataset composition, statistical profiles, annotation methodologies, and data partitioning protocols is provided in Section 3.2.3. Requests for access to supporting evaluation protocols and processed experimental results should be directed to the corresponding author upon reasonable request, subject to confidentiality obligations and pending completion of requisite data usage formalities and approval from the competent authority of CARDC.

## Abbreviations

The following abbreviations are used in this manuscript:

AWIE	Adaptive Wavelet Image Enhancement
CGAF	Cross-Gated Attention Fusion
IGU	Independent Gating Unit
IR-D	infrared–depth
MFI	Multimodal Feature Interaction
